# Revolutionizing Cardiovascular Frontiers: A Dive Into Cutting-Edge Innovations in Coronary Stent Technology

**DOI:** 10.1097/CRD.0000000000000705

**Published:** 2024-05-06

**Authors:** Alice Valeria Wiyono, Azizah Puspitasari Ardinal

**Affiliations:** * Faculty of Life Sciences & Medicine, King’s College London, School of Cardiovascular and Metabolic Medicine, London, United Kingdom.

**Keywords:** coronary stent, drug-eluting stents, coronary artery disease, coronary intervention

## Abstract

Plain balloon angioplasty was the initial method used to enlarge the intracoronary lumen size. However, it was linked to acute coronary closure due to early vessel recoil. This led to the invention of coronary stents, which offer mechanical support to open and maintain the vascular lumen. Nevertheless, the metallic scaffold introduced other issues, such as thrombosis and restenosis caused by neointimal proliferation. To address these concerns, polymers were employed to cover the scaffold, acting as drug reservoirs and regulators for controlled drug release. The use of polymers prevents direct contact between blood and metallic scaffolds. Drugs within the stent were incorporated to inhibit proliferation and expedite endothelialization in the healing process. Despite these advancements, adverse effects still arise due to the inflammatory reaction caused by the polymer material. Consequently, resorbable polymers and scaffolds were later discovered, but they have limitations and are not universally applicable. Various scaffold designs, thicknesses, materials, polymer components, and drugs have their own advantages and complications. Each stent generation has been designed to address the shortcomings of the preceding generation, yet new challenges continue to emerge. Conflicting data regarding the long-term safety and efficacy of coronary stents, especially in the extended follow-up, further complicates the assessment.

The treatment of coronary artery disease has been completely transformed with the introduction of coronary angiography. Plain old balloon angioplasty (POBA) without stent implantation was the first procedure used to enlarge intracoronary lumen size and was first identified in 1977.^1^ However, POBA is associated with an increased risk of acute artery closure and restenosis, which may be brought on by early vessel recoil or dissection, neointimal proliferation, and late vascular healing.^2^

Bare metal stent (BMS) was developed in 1987, and it showed less stenosis than POBA.^3,4^ BMS features a scaffolding structure that minimizes recoil and helps to close the dissection flaps. Still, there were several drawbacks, including subacute stent thrombosis (ST) due to the high metallic components, deployment failure due to the thick strut design, and substantial risk of in-stent restenosis (ISR).^3–5^

Another revolution in 2003 was the introduction of drug-eluting stents (DES). DES, a kind of BMS coated with antiproliferative medicines like sirolimus and paclitaxel, brought a significant ISR reduction.^6–8^ The polymer that covers DES serves as a drug reservoir and a barrier between blood and metal surfaces. The initial generation of DES raised concern regarding late and very late ST. Second-generation DES was constructed with thinner struts, improved scaffold designs, and different drugs and polymers utilized, yet the concern of polymer durability persists. The third-generation biodegradable polymer (BP) DES addressed the issue. More sophisticated stent technology without polymers was invented. The latest breakthrough is the bioresorbable scaffold (BRS), which aims to solve the consequences of scaffold persistence. Further understanding of stent structure and design modifications is necessary to improve safety and efficacy. Using the evolution of coronary stents as a guide, this review is intended to shed light on the prospective stent innovation.

## BARE METAL STENTS

The first US food drug and administration (FDA)-approved BMS was introduced in 1987; it is composed of stainless steel (SS) since its high elastic modulus and excellent tensile strength give adequate radial force to open the vessel and minimize recoil.^9–11^ In 1994, the FDA-approved using BMS to address acute and threatened closure following unsuccessful percutaneous transluminal coronary angiography procedures.^12^ BMS was designed with thick struts to preserve radial force and radio-opacity. Unlike POBA, BMS remains in the lesion area, maintaining coronary arterial patency through early recoil prevention. However, it carried a significant risk of subacute ST due to the stent’s dense metallic content and thick strut design, which also challenged its deployment and caused embolization.^13^ The exposed metallic surface acts as a focal point for platelet aggregation and contributes to plaque instability through chronic inflammatory response, which primarily involves macrophages. Additionally, matrix degradation by metalloproteinases can further destabilize the plaque.^14–16^ Despite a lower rate of restenosis than POBA (32% vs 42%), there was still a substantial risk of restenosis.^3^ Stent implantation causes vascular damage, stimulates fibroblasts and smooth muscle cells (SMCs) in the inflammation process, and leads to neointimal hyperplasia. There were additional concerns over radio-opacity and incompatibility with magnetic resonance imaging due to the dense metallic content. To improve X-ray visibility, gold was added, which regrettably raised the risk of ISR and death.^17,18^

On the other hand, some investigations proved that the incidence of myocardial infarction (MI) and mortality rate are comparable in individuals treated with either BMS or DES.^19–21^ Data over the past decade indicates that BMS is utilized in 20% of coronary angioplasty procedures,^22^ as well as procedures involving greater vessels because of the lower restenosis rate.^23^ The ongoing use of BMS can be attributed to factors such as cost-effectiveness and the possibility of shortening the duration of dual antiplatelet therapy (DAPT) after the intervention, particularly in the high bleeding risk populations such as the elderly.^24^

Despite the 2021 ACC/AHA/SCAI guideline for coronary artery revascularization prioritizing DES over BMS is recommended to mitigate the risks of ISR, ST, and MI, there are situations where BMS may still be considered. This includes circumstances where DES is unavailable or when patient conditions necessitate very brief DAPT (<1-month). Moreover, for patients with stable ischemic heart disease, the guideline suggests DAPT following BMS-percutaneous coronary intervention, with a 1-month duration recommended as part of class I recommendations. Conversely, for those undergoing DES-percutaneous coronary intervention, a 6-month DAPT regimen is advised, with the option of aspirin cessation after 1 to 3 months and transitioning to P2Y12 monotherapy.^25^

Furthermore, design and material innovations in BMS have advanced in recent years. In the OMEGA study, platinum-chromium (PtCr) BMS demonstrated satisfactory safety and efficacy in treating coronary lesions in early and 12-month post-angiographic intervention. Boston Scientific Corporation markets this coronary stent as the REBEL stent; it was designed with a thin strut (81 µm) and extra connections within the proximal end of the stent to maximize tensile strength while preserving deliverability.^26^ Thinner struts are known to have lower neointimal proliferation and greater endothelialization capability than thicker struts, possibly due to less vascular damage during the procedure.^27–30^ Other examples of currently used coronary BMS are Vision, Driver, and Coroflex Blue, which are composed of cobalt-chromium (CoCr).^21^

## FIRST-GENERATION DRUG-ELUTING STENTS

Although successfully preventing early recoil and abrupt lumen closure,^2^ BMS also induces neointimal proliferation due to vascular injury, causing SMC proliferation and ultimately resulting in ISR.^12^ Therefore, it is essential for the stent to elute medications that alleviate inflammation and inhibit the proliferation of neointimal cells. DES prevents restenosis since it can elute an antiproliferative agent and has a carrier that regulates drug release.^31^ Antiproliferative medications such as Sirolimus and Paclitaxel are utilized in the case. Sirolimus inhibits the mammalian target of the rapamycin receptor, hence decreasing vascular SMC proliferation by suppressing the G1-to-S phase transition of the cell cycle.^29^ Paclitaxel suppresses cell proliferation by stabilizing microtubule polymer to prevent its disassembly, and it is extensively utilized as a breast cancer treatment.^12,32,33^ Since both drugs have a relatively short half-life, a carrier, which is a polymer, was added to regulate drug release, consequently maintaining the drug over a prolonged duration, thus preventing restenosis.^34,35^ Conversely, studies revealed that restenosis rates were not improved to a clinically tolerable range by modifying metallic components, stent design, or drug coatings including heparin, although heparin successfully lowered ST.^17,32^

The Cypher sirolimus-eluting stent (SES) was released in 2002 as the first-generation DES. Two years later, a second first-generation DES called Taxus paclitaxel-eluting stent (PES) was invented.^36^ Both Cypher and Taxus were covered with durable and non-BP: polyethylene-*co*-vinyl acetate and poly-*n*-butyl-methacrylate in the Cypher SES; poly (styrene-*b*-isobutylene-*b*-styrene) in the Taxus.

Taxus PES was tested in a number of different clinical studies, showing its superiority over BMS in both safety and efficacy.^37–40^ However, within 1 or 2 years of follow-up, the SIRIUS and RAVEL trial demonstrated that SES outperformed other types of DES, including PES.^41,42^ This is likely because of the superior coating technology of SES that affects the drug kinetics, causing faster drug release from Cypher compared to Taxus—takes years to release 90% of the drug. Ideally, the drug should be released immediately to reduce restenosis and thrombosis, later on, gradually slowed to promote endothelialization.^36^ Conversely, the REALITY trial has shown that both SES and PES have the equivalent major adverse cardiovascular events (MACE) rate, but PES had greater ST risk, whereas SES had less ISR risk.^43^

Despite its superiority over BMS, the first-generation DES was linked with complications such as delayed endothelialization, prolonged inflammation, and enhanced likelihood of ST.^19,44^ Polymer components are the reason behind inflammation and delayed hypersensitivity reactions. Moreover, the incorporated drugs cause delayed endothelialization and early neoatherosclerosis.^35,45–47^ In the porcine model, the Cypher was superior in lowering neointimal growth at 28 days compared to BMS; however, after the drug had been entirely released, which is at 90 to 180 days, inflammation, proliferation, and delayed endothelialization were observed.^48^ All of these are related to the polymer persistence. Whereas, thick strut design prompted issues over thrombogenicity and reduced endothelialization rate.^49,50^

## SECOND-GENERATION DRUG-ELUTING STENTS

The second-generation DES was built with thinner struts, a redesigned scaffold architecture, newer drug kinds, and better polymer covering than its predecessor.^51^ Both ISAR-STEREO and ISAR-STEREO 2 trials showed improved stent quality and reduced stent-related complications, including mortality rates in patients who received thinner struts instead of thicker struts.^29,30^ Because thinner struts have a smaller contact area, they minimize vascular damage, inflammation, neointimal proliferation, and thrombosis and speed up endothelialization, therefore displaying improved clinical outcomes.^52^ Other benefits include improved deliverability and provide a wider stent inner diameter, which also prevents injury during implantation.^9,10,13^ To maintain radial force and tensile strength despite the thinner strut design, CoCr and PtCr alloys were utilized due to their greater density, yield strength, tensile strength, and elastic modulus than SS.^53^

Everolimus and zotarolimus, which are both derivatives of sirolimus, were introduced as part of the second-generation of DES. The finest second-generation DES are those coated with everolimus. In comparison to sirolimus, it is lipophilic, has a polarity, superior bioavailability, excellent absorption, shorter clearance time, and a longer drug-target residence time.^54,55^ Zotarolimus is the analogue of sirolimus that is the most hydrophobic and lipophilic; hence, it is released slowly and persists.^56^ By assessing definite/probable ST, diameter, and bifurcations, the ESTROFA-2 study comparing zotarolimus-eluting stent (ZES) and everolimus-eluting stent (EES) revealed that ZES is comparable to EES in terms of thrombogenicity compared to older generation stents. However, in patients with bifurcation lesions, ZES may pose a higher risk of ST.^57^ Moreover, ZES was shown to be noninferior to EES in the Resolute All Comers study with regard to the primary endpoint: composite of cardiac mortality, MI, or clinical indication of target lesion revascularization (TLR) within 12 months; ISR; ST; and late lumen loss (LLL).^58^ A longer-term study observed 2-year clinical outcomes from the Resolute All Comers trial and revealed similar safety and efficacy between ZES and EES as in the 1-year follow-up.^59^ Several clinical trials (Table 1) have shown that second-generation EES was either noninferior or superior to first-generation PES or SES. Conversely, it was demonstrated that second-generation ZES was inferior to first-generation SES, particularly in the short-term follow-up. No differences in terms of TLR and ST in the long-term follow-up. Fortunately, it was still superior to first-generation PES. Therefore, it can be concluded that although second-generation ZES and EES have comparable effectiveness, and both are superior to PES, the second-generation EES, such as Xience, is regarded as the safest of the 3 since it has been found to minimize ST and MI occurrence.

**TABLE 1.** Safety and Efficacy Studies of the Second-Generation DES

Studies (Year)	Stents	Follow-Up Period	Outcomes
ENDEAVOR III^60,61^ (2006)	Endeavor ZES vs Cypher SES	9 months	MACE 0.6% vs 3.5% LLL 0.34 ± 0.44 mm vs 0.13 ± 0.32 mm Restenosis 11.7% vs 4.3% TLR 9.8% vs 3.5%
		5 years	All-cause mortality 5.2% vs 13.0% MI 1.0% vs 4.6% Composite cardiac mortality and MI 1.3% vs 6.5% MACE 14.0% vs 22.2% Progression of MACE from 9 months to 5 years 7.8% vs 16.7% No differences in TLR and ST
SPIRIT II^62,63^ (2006)	Xience V EES vs Taxus Liberte PES or Taxus Express PES	6 months	LLL 0.11 ± 0.27 mm vs 0.36 ± 0.39 mm In-stent volume obstruction 2.5 ± 4.7% vs 7.4 ± 7.0% MACE 2.7% vs 6.5%
		5 years	Cardiac mortality 1.5% vs 7.3% TLR 4.7% vs 9.4% MACE 8.0% vs 18.1% ST 0.9% vs 2.8%
SPIRIT III^64,65^ (2008)	Xience V EES vs Taxus Express PES	9 months	LLL 0.14 ± 0.41 mm vs 0.28 ± 0.47 mm TVF 7.2% vs 9.0% MACE 4.6% vs 8.1%
		12 months	MACE 6.0% vs 10.3%
		3 years	Primary endpoint of TLF 13.5% vs 19.2% MACE, MI, cardiac mortality, ischemic-driven TLR 9.1% vs 15.7%
ESTROFA-2^57^ (2010)	ZES vs EES	2 years	No difference in definite/probable thrombosis Similar increment of definite thrombosis Higher thrombosis rate in ZES-patient with bifurcations (HR 4; 95% CI, 1.1–1.3)
Resolute All Comers^58^ (2010)	Resolute ZES vs Xience V EES	13 months	TLF (cardiac mortality, TV-MI, TLR) 8.2% vs 8.3%; significant *P* value for noninferiority No differences in cardiac mortality, MI, revascularization, ST, LLL, and adverse events
SORT OUT III^66,67^ (2010)	Endeavor ZES vs Cypher SES	9 months	Primary endpoint 6% vs 3% No difference in all-cause mortality
		18 months	Primary endpoint 10% vs 5% All-cause mortality 4% vs 3%
		3 years	MACE 12.9% vs 10.1% TLR 9.1% vs 6.7% Very late definite ST 0% vs 1.1% No differences in MI and cardiac mortality
ENDEAVOR IV^68,69^ (2010)	Endeavor ZES vs Taxus Express PES	9 months	TVF 6.6% vs 7.1%, significant *P* value for noninferiority MI 0.5% vs 2.2%
		12 months	No differences in ST, TLR, MI, and cardiac mortality
		5 years	MI or cardiac mortality 6.4% vs 9.1% Very late ST 0.4% vs 1.8% Late MI 1.3% vs 3.5% No differences in ST, TLR, TVF
ZEST^70^ (2010)	Endeavor ZES vs Cypher SES or Taxus Liberte PES	12 months	MACE 10.2% vs 14.1% No differences in MI and death ST 0.7% vs 0% vs 0.8%
COMPARE^71,72^ (2010)	Xience V EES vs Taxus PES	12 months	Primary endpoint 6% vs 9%; significant *P* value for superiority ST <1% vs 3% MI 3% vs 5% TLR 2% vs 6% Cardiac mortality, nonfatal MI, or TLR 5% vs 8%; significant *P* value for superiority
		2 years	Composite death, nonfatal MI, and TLR 9.0% vs 13.7% MI 3.9% vs 7.5% TLR 3.2% vs 8.0% Definite/probable ST 0.9% vs 3.9%
SPIRIT IV^73,74^ (2011)	Xience V EES vs Taxus Express PES	12 months	Primary endpoint of TLF 4.2% vs 6.8% Ischemic-driven TLR 2.5% vs 4.6% ST 0.17% vs 0.85% MI 1.9% vs 3.1%
		2 years	TLF 6.9% vs 9.9% Ischemic-driven TLR 4.5% vs 6.9% MI 2.5% vs 3.9% Q-wave MI 0.1% vs 0.8% ST 0.4% vs 1.2%
EXCELLENT^75^ (2011)	Xience V EES or Promus vs Cypher SES	9 months	In-segment LLL 0.11 ± 0.38 mm vs 0.06 ± 0.36 mm In-stent LLL 0.19 ± 0.35mm vs 0.15 ± 0.34 mm No differences in TLF and ST
SORT OUT IV^76^^–^^78^ (2012)	Xience V EES vs Cypher SES	9 months	Primary endpoint 4.9% vs 5.2%; significant *P* value for noninferiority Definite ST 0.1% vs 0.7%
		18 months	Primary endpoint 7.2% vs 7.6% Definite ST 0.2% vs 0.9%
		2 years	Primary endpoint 8.3% vs 8.7% Definite ST 0.2% vs 0.9% No differences in patient-related outcomes and stent-related outcome
		5 years	MACE 14.0% vs 17.4% Definite ST 0.4% vs 2.0%
RESET^79^ (2012)	Xience V EES vs Cypher SES	12 months	Primary endpoint of TLR 4.3% vs 5.0%; significant *P* value for noninferiority In-segment LLL 0.06 ± 0.37 mm vs 0.02 ± 0.46 mm; significant *P* value for noninferiority No difference in definite ST
PROTECT^80^ (2014)	Endeavor ZES vs Cypher SES	4 years	Primary outcome 1.6% vs 2.6% All-cause mortality or MI 6.7% vs 8% No difference in DAPT treatment

EES, everolimus-eluting stent; LLL, late lumen lose; MACE, major adverse cardiovascular events; PES, paclitaxel-eluting stent; SES, sirolimus-eluting stent; ST, stent thrombosis; TLF, target-lesion failure; TLR, target lesion revascularisation; TV-MI, target vessel myocardial infarction; ZES, zotarolimus-eluting stent.

In response to the polymer durability issues reported in the first-generation of DES, more innovative polymers were developed: poly(vinylidene fluoride-*co*-hexafluoropropylene) (PVDF-HFP)—utilized by Xience EES, with the ability to withstand expansion while preserving elongation elasticity,^49^ and the extremely hydrophilic phosphorylcholine polymer component that does not stimulate inflammation.^81^ However, Byrne et al^75^ still observed late and very late ST, late inflammation, and delayed endothelialization, all of which were related to polymer persistence despite the fact that the drugs had been entirely released. Subsequently, this polymer drives inflammation and regenerates the process of restenosis.

## THIRD-GENERATION DRUG-ELUTING STENTS

Earlier generations of DES have been proven to increase the chance of very late ST, necessitating longer DAPT treatment. Polymeric coatings can elicit inflammatory responses in the lesion, increasing the chances of thrombosis and delayed endothelialization. Endothelial dysfunction arises from exposure of the artery wall to sirolimus or its analogues and is responsible for delayed healing and neoatherosclerosis. The third-generation DES was developed to address the shortcomings of previous generations, primarily by utilizing polymers that are more biocompatible. Furthermore, the invention of polymer-free DES diminishes the possibility of polymer-induced inflammatory stimuli.

BP DES is characterized by regulated drug elution alongside polymer degradation in the hydrolytic circumstance. Only BMS remains after the drug has been released and the polymer has been dissolved.^82^ Polylactic acid, polyglycolide, poly-d,l-lactic acid, and poly(D,L-lactic-*co*-glycolic acid) (PLGA) are the most frequently used polymers in the BP DES. In polymer-free DES, the drug is embedded onto the microporous or nanoporous stent surface, ultimately minimizing long-term complications of polymer persistence and enhancing vascular endothelialization and stent integrity. A wide variety of drugs from all preceding generations are utilized, with additional new analogues of sirolimus named biolimus, a highly lipophilic drug. In the same way as sirolimus does, biolimus possesses anti-inflammatory and antiproliferative properties and an improved elution timescale.^36^ Biolimus is utilized in BioFreedom DES.

Types of metallic components used are the same as the first- and second-generation DES: SS, CoCr, and PtCr. PtCr is solely utilized for BP stents. However, since SS and CoCr offer a wider variety of surface variations, thus having the capacity to retain the drug without the need for polymer, and they can be utilized in both BP DES and polymer-free DES.^36^ Short- and long-term clinical studies revealed the noninferiority of third-generation DES over second-generation DES, as well as the superiority over BMS and first-generation DES.^83–93^

An additional decrease in strut thickness (<70 µm) was also accomplished; these devices are known as ultrathin strut DESs, and they enable improved deliverability, less damage to the vessel walls, and less occlusion of small arterial branches. For instance: Orsiro BP SES has a strut thickness of 60 µm in stents with a diameter of 2.25 to 4 mm, BioMime BP SES has 65 µm, MiStent BP SES 64 µm, and Supraflex BP SES 60 µm.^94^ BIOFLOW I-V trials comparing Orsiro BP SES and Xience showed improved LLL, lower target vessel myocardial infarction (TV-MI), target-lesion failure (TLF) risk and mortality, with the Orsiro than Xience in the short- and mid-term follow-up.^95–99^ BIOSCIENCE study also proved the noninferiority of Orsiro over Xience 12-month postimplantation. Unfortunately, at a 5-year follow-up, there were no differences in TLF between both stents.^90,91^ According to SORTOUT VII and IX studies, Orsiro was related to a decreased definite/probable ST and TLF over Nobori and BioFreedom.^100,101^ This is reasonable since both have a thicker SS-strut than the Orsiro.

The noninferiority of thin strut third-generation DES over the previous generations in the short-term period was also observed in MiStent BP SES. MiStent was built with PLGA polymers which are reabsorbed within 3 months, thus lowering the inflammatory response. It has microcrystals to allow a regulated drug release. Dessolve I trial showed a low and stable LLL, complete strut coverage, and no ST following 18 months after MiStent implantation. Dessolve II trial revealed no difference in TLF with the use of MiStent or Endeavor for a 5-year follow-up period. Whereas, Dessolve III trial observed the noninferiority of MiStent compared to Xience in terms of cardiac mortality, TV-MI, and TLF within 12 months of follow-up.^102–105^ TALENT trial also demonstrated the noninferiority of CoCr Supraflex BP SES versus Xience in terms of cardiac mortality, TV-MI, and TLR over the short-term period, and confirmed its continued safety and efficacy in the 2nd year.^106,107^

Therefore, ultrathin strut BP DES may be beneficial only in the short-term because the benefits diminish after the complete breakdown of polymers and complete endothelialization. Additionally, ultrathin strut stents may find it difficult to expand in calcified lesions or chronic total occlusion when a strong radial force is needed. Ultrathin struts have less stent expansion capability, making their application in larger arteries complicated.^108^ Hence, when considering the use of ultrathin strut stents in challenging scenarios like these, incorporating lesion preparation strategies becomes indispensable for optimal stent deployment and favorable long-term outcomes. Notably, calcium modification techniques, such as athero-ablative atherectomy, involve employing rotational or orbital atherectomy devices to remove plaque or calcium deposits within the vessel wall. This process creates a wider, smoother luminal surface conducive to stent deployment and expansion, thereby enhancing blood flow. Additionally, balloon-based techniques like cutting and scoring balloons, which exert higher pressure against arterial walls and create incisions in the plaque, aid plaque modification and vessel expansion. These approaches are particularly useful in disrupting calcified or fibrotic lesions and can be employed to facilitate stent expansion and apposition in calcified lesions, ultimately aiming to alter plaque composition, reduce calcium burden, and enhance vessel compliance.

More advanced DES without polymer may solve the consequence of complete polymer breakdown. A nonpolymer DES has micro or nanopores to store the drug. Coroflex ISAR Neo is an ultrathin strut polymer-free CoCr SES with probucol to control drug elution. Probucol is a highly lipophilic matrix builder with antioxidant and lipid-lowering properties. In the short-term follow-up, ISAR TEST 5 proved the noninferiority of Coroflex over Resolute ZES and showed low definite/probable ST occurrence in predetermined age, gender, diabetic status, and vessel diameter groups in 5 to 10 years.^109–111^

## BIORESORBABLE SCAFFOLD

BRS was invented after BP’s success. Unfortunately, no 100% of the BRS on the market is FDA-approved at the moment. BRS addresses scaffold persistence issues such as compromised coronary vasomotor function, chronic inflammation due to retained foreign-body metal, and very late ST due to uncovered struts. After completely resorbed, it is expected that the coronary artery will recover to its normal condition, thereby limiting cardiac events, allowing discontinuation of DAPT, and permitting coronary bypass surgery if necessary. The incorporation of radio-opacity markers promises noninvasive imaging for evaluation. In addition, it minimizes long-term obstruction of side branches. BRS was built with biodegradable materials such as polymer and magnesium (Mg). Several polymeric and metallic compounds have been evaluated, and the preliminary findings with poly-*L*-lactide (PLLA)–used in Orsiro as well, are encouraging.^112–116^ However, the polymer has weaker radial force than metallic substances such as SS, PtCr, CoCr and Mg, thus requiring a thicker strut design.

### Polymer-Based Bioresorbable Scaffold

#### Absorb

Abbott’s Absorb bioresorbable vascular scaffold (BVS), the first BRS, made up of 156 µm PLLA scaffold that completely degraded within 3 years and does not elute drug. A 10-year follow-up study revealed the long-term safety of the PLLA stent in terms of MACE and scaffold thrombosis.^117^ Throughout the years, ABSORB trials concluded the inferiority of Absorb BVS over Xience EES in terms of TLF and ST.^113,118–121^ Dudek et al^122^ discovered similar vasomotor responses between patients treated with Absorb BVS and DES, suggesting Absorb BVS is ineffective for restoring normal endothelial function. Consequently, Absorb BVS was withdrawn from the market.

#### DESolve

DESolve from Elixir Medical is a balloon-expandable novolimus-eluting PLLA-based BRS with 150 µm strut. This stent degrades and resorbs rapidly, with >90% degraded at 6 months and 70% resorbed at 12 months. It has a wider expansion range and is capable of resolving minor malposition through a self-correction function following early recoil.^123,124^ The new DESolve model expects 95% biodegradation in 1-year and full resorption in 2 years.^125,126^ Following the complete degradation and resorption, proteoglycans replace polymer and build connective tissues.^125^ DESolve Nx trial revealed good safety and efficacy profiles of DESolve up to 2 years, which are 3.3% of MACE at 6 months and 7.4% at 2 years, significant increase of lumen and scaffold diameter at 6 months, and 99% of full strut coverage.^126^ In line with this, the 12-month DESolve PMCF study demonstrated significant in-scaffold lumen expansion and lower stenotic diameter, and only 1% ST and 3% MI with zero cardiac mortality.^127^ Both the DESolve Nx and PMCF studies observed earlier and greater in-scaffold lumen expansion with the DESolve scaffold compared to the Absorb device.^128,129^ Despite employing the same PLLA polymer, it seems that DESolve outperformed Absorb BRS in terms of performance and short-term safety, most likely due to its pro-remodeling property after resorption, self-correction capacity, greater fracture resistance to massive stretching, and rapid degradation and resorption. Short degradation time is beneficial to prevent late and very late ST, as well as scaffold disintegration. However, validation of the long-term safety of the device should be done.

#### MeRES100

MeRES100 by Meril Life Sciences is the first 100 µm thick sirolimus-eluting PLLA-based scaffold. This scaffold degrades within 2 to 3 years. MeRes-1 trial revealed good safety and performance of MeRes100 in treating de novo coronary lesions by showing only 0.93% MACE and zero ST, 11.33% in-scaffold stenosis, and full integrity of the scaffold within 1-year, 99% neointimal strut coverage and low neointimal hyperplasia within 6 months.^130^ A 3-year clinical follow-up revealed no ST and a 2-year imaging follow-up revealed maintained lumen patency, indicating continued safety and performance.^131^ MeRes100’s success is a result of its improved architecture, which features open cells in the middle for better deliverability, a wider expansion range, and fracture prevention, and closed cells at both edges for better coverage and to prevent embolization; a thinner strut to limit inflammation and neointimal proliferation; and rapid degradation.

### Magnesium-Based Bioresorbable Scaffold

Biotronik Inc. introduced a magnesium-based Magmaris open-cell scaffold. They promise stronger radial strength and lesser thrombosis than Absorb BVS. The 150 μm thick strut stent has sirolimus-eluting PLLA coating. 95% of Mg-based stents are resorbed in 12 months, with Mg eliminated through the kidney, the strut replaced by a compound of calcium phosphate, and PLLA dissolved by 2 years.^132,133^ This fast resorption can prevent ST. Ex vivo models have demonstrated that magnesium ions may have antithrombotic properties due to reduced inflammatory cell and platelet accumulation.^134^ Similar to bioresorbable polymer-based stents, however, such thick struts damage the artery during implantation, increase the thrombosis area, and demand larger catheters which makes their delivery difficult. Whilst outperforming Absorb BVS, the Magmaris does not satisfy the specific design criteria proposed by The Minerals, Metals, and Materials Society for the biodegradable stent, including tensile strength >300 MPa, yield strength >200 MPa, and elongation to failure >20–30%.^135–137^ It is also degrading rapidly, which is 50 to 100% quicker than expected.^136^ However, once zinc and manganese are added, the material strength is comparable to SS stents, with less than 8% elastic recoil, less than 5% shortening after inflation, and elevated collapse pressures.^138^

The progression of Mg-based stents apparently started with Biotronik’s AMS 1 stent. This stent has a bulky structure that limits its deliverability, and poor radial force. According to the PROGRESS AMS trial, this stent was at high risk for TLR (23.8%) and restenosis (47.5%) in 4 months of follow-up, despite reducing in-stent stenotic diameter from 61.48% (SD 13.10) to 12.65% immediately (SD 5.63). Nonetheless, at a 4-month follow-up, the stenotic diameter had increased again to 48.37%. (SD 17.00). Several advantages were discovered, including decreased in immediate recoil, reduced hyperplasia, zero ST, MI, and cardiac mortality, and safe degradation within 4 months. Thus, proving a great safety profile of this stent, but not satisfactory on its performance.^138^ Therefore, it was required to decelerate the scaffold degradation process and inhibit cell proliferation.

Due to the high TLR and restenosis rate of the AMS 1 stent, Biotronik developed the DREAMS 1G stent. This balloon-expandable scaffold is coated with PLGA and paclitaxel, which inhibits neointimal proliferation. Paclitaxel elution is within the first 3 months along with the PLGA degradation, whereas the scaffold itself is degraded within 9–12 months. Moreover, DREAMS 1G was designed with better alloy compound and strut configuration to improve scaffolding abilities.^139^ Patients treated with DREAMS 1G had a low TLF rate and no cardiac mortality or MI after 6 months, 1-year, and 3 years of follow-up in the BIOSOLVE I study. Despite the good safety profile, findings still revealed a significant LLL which suggests its inferiority to those of contemporary DES.^115,138,140^ DREAMS 2G, the successor to DREAMS 1G, is a balloon-expandable SES with PLLA coating; this device is also known as the Magmaris scaffold. DREAMS 2G shown an improvement in LLL whilst preserving performance and safety. DREAMS 2G’s scaffold is more flexible and durable, resulting in greater radial force and deliverability. It also employed sirolimus and PLLA, the same coating utilized by the successful Orsiro, to be more efficient in lowering neointimal proliferation.^96^ In BIOSOLVE II trial, DREAMS 2G demonstrated a similar vasomotor response with DREAMS 1G, better degradation within 6 months, reduction of neointimal hyperplasia, LLL, TLR, and TLF, zero definite/probable ST, and vasomotor function restoration in 6 months.^96^ Similar safety and efficacy were also seen in 12 months of follow-up.^141^ However, there was a slight rise in LLL and stenosis between 12 and 36 months.^142^

Despite those benefits, all studies were conducted on simple de novo lesions, therefore only recommended in patients with stable angina. Additionally, lack of data in subgroup of patients with ST-elevation MI, routine DAPT, or diabetes. Whereas, the thick strut design may induce inflammation and platelet activation. Comparison between each generation of marketed coronary stents can be seen in Table 2.

**TABLE 2.** Comparison Between Each Generation of Coronary Stents

Generation	Years (Developed or Approved)	Name	Manufacturer	Drug	Drug Release Kinetics	Polymer	Indication	Contraindication
BMS	1987	Palmaz-Schatz	Johnson & Johnson	–	N/A	–	Symptomatic IHD	Allergy to metal alloy
	FDA: 1993	Gianturco-Roubin	Cook Inc.	–	N/A	–	Symptomatic IHD	Allergy to metal alloy
First-generation DES	FDA: 2003	Cypher	Cordis Corp.	Sirolimus	Rapid release, peak <30 days	Durable	De novo coronary lesions	Allergic to drug or polymer
	FDA: 2004	Taxus	Boston Scientific	Paclitaxel	Moderate release, peak <90 days	Durable	De novo coronary lesions	Allergic to drug or polymer
	FDA: 2008	Endeavor	Medtronic	Zotarolimus	Slow release	Durable	De novo and restenotic lesions	Allergic to drug or polymer
Second-generation DES	FDA: 2008	Promus	Boston Scientific	Everolimus	Steady release, 90–120 days	Durable	De novo and restenotic lesions	Allergic to drug or polymer
	FDA: 2011	Xience V	Abbott	Everolimus	Steady release, 90–120 days	Durable	De novo and restenotic lesions	Allergic to drug or polymer
	FDA: 2012	Resolute Integrity	Medtronic	Zotarolimus	Controlled release, >180 days	Durable	De novo and restenotic lesions	Allergic to drug or polymer
Third-generation DES	FDA: 2015	Synergy	Boston Scientific	Everolimus	Controlled release, 120 days	Bioabsorbable	De novo, complex lesions, restenotic lesions and diabetic patients	Allergic to drug or polymer
	FDA: 2019	Orsiro	Biotronik	Biolimus	Controlled release, <6 months	Bioabsorbable	De novo, restenotic lesions	Allergic to drug or polymer
	CE Mark: 2013	Ultimaster	Terumo	Sirolimus	Controlled release, 90 days	Bioabsorbable	De novo coronary lesions	Allergic to drug or polymer
BRS	CE Mark: 2011 FDA: 2016 Withdrawn: 2017	Absorb 1.0	Abbott	Everolimus	Controlled release, 120 days	Fully bioresorbable scaffold	De novo coronary lesions	Allergic to drug or polymer, scaffold integrity
	CE Mark: 2013	DESolve Nx	Elixir Medical	Novolimus	Controlled release, <4 months	Fully bioresorbable scaffold	De novo coronary lesions	Allergic to drug or polymer, scaffold integrity
	CE Mark: 2016	Magmaris	Biotronik	–	N/A	–	De novo coronary lesions	Allergic to magnesium alloy
	CE Mark: 2019	MeRes100	Meril Life Sciences	Sirolimus	Controlled release, 90 days	Fully bioresorbable scaffold	De novo coronary lesions	Allergic to drug or polymer, scaffold integrity

BMS, bare metal stent; DES, drug-eluting stent; BRS, bioresorbable scaffold.

## POLYMER COATINGS

Polymer coatings serve multiple purposes on stents, including as a drug reservoir and regulating drug release. In regulating drug delivery kinetics, as seen in DES, polymers exert control over the rate and duration of drug release, ensuring an optimal therapeutic outcome whilst mitigating systemic exposure and adverse effects. Given that vascular SMCs initiate hyperproliferation within 1 day following stent implantation, it is essential to administer antirestenotic medications for a minimum of 3 weeks postimplantation to inhibit SMC proliferation and migration.^143^ Incorporating anti-inflammatory drugs into polymer-coated DES can ameliorate inflammatory response. Additionally, biocompatible polymers with smooth surfaces and controlled degradation profiles serve to diminish inflammatory responses and augment tissue compatibility.

However, polymers also impede the progression of endothelialization. The first-generation DES had certain drawbacks, which, owing to the polymer components used in the device, were associated with inflammation and delayed hypersensitivity reactions. It contains durable polymers that have been demonstrated to trigger a thrombogenic response as a result of the inflammatory process, delayed healing, and inadequate endothelialization.^144,145^ Additionally, the drugs integrated into the DES led to delayed endothelialization and early neoatherosclerosis.^35,45–47^ The ideal drug dosage and release kinetics would, therefore, be those that effectively hinder SMC proliferation while preserving endothelialization.^146^ Moreover, polymer coatings may elicit inflammatory responses, potentially influencing thrombogenicity and restenosis rates. This results from factors such as polymer degradation products, surface irregularities, or foreign-body reactions to the stent material. Significant advancements in polymer technology have led to the development of more biocompatible and durable coatings. Commonly utilized polymer materials encompass biodegradable options such as PLGA, poly(ethylene glycol), and polylactic acid, alongside nonbiodegradable alternatives such as polyethylene-*co*-vinyl acetate and polyurethane.

Comprehending the kinetics of drug delivery is essential, as swift drug release may surpass tissue absorption, whilst a slow pace release could hinder endothelialization.^147^ The drug release mechanism is expected to be slow, gradual, and regulated, wherein the antiproliferative drug can be promptly delivered within the first week, but the overall release duration should be sustained for a period of 1 to 3 months following stent implantation.^148,149^ Furthermore, another significant challenge encountered with the first-generation DES was the issue of inadequate drug delivery.^150^ However, the release of large amounts of drugs at the beginning exposes the tissue to the polymer, which is known to induce inflammation, thereby slowing down the healing process and reducing endothelialization.^147,151^ High drug concentrations that overpower tissue receptors can result in thrombosis, medial necrosis, and intimal bleeding. These conditions are linked to ST.^152,153^ Both durable and biodegradable DES are linked to initial rapid release.^154^

## CONCLUSION

The advancement of stent technology has yielded better clinical outcomes, such as lower thrombosis and restenosis rates and accelerated healing. Each generation has been designed to address the shortcomings of the preceding generation, yet new issues continue to emerge. So far, several BMS are still marketed for use in patients whose DAPT duration must be shortened. Xience and Orsiro are examples of commonly used DES due to their favorable outcomes. BRS, the most recent innovation that ingeniously adapted Xience and Orsiro technologies, is expected to alleviate polymer-related and stent retention concerns. Despite providing the best performance and safety, no 100% of BRS on the market is FDA-approved at the moment. Moreover, the advanced BRS are only used in noncomplex de novo lesions and limited study of subgroup populations. Future innovation should emphasize BRS materials that provide stronger radial force with thinner strut design, thereby minimizing inflammatory response and restenosis, accelerating healing, restoring vasomotion, and reducing recoil. The performance of the scaffold should be comparable to those of metallic DES. In addition, a long-term follow-up study and subgroup analysis of BRS is necessary.
